# Effects of hydrolysed meat on dietary intake and nutritional status in aged care residents requiring pureed diets: a crossover randomised controlled trial

**DOI:** 10.1186/s12877-022-03622-2

**Published:** 2022-11-25

**Authors:** Xiaojing Sharon Wu, Anna Miles, Andrea Braakhuis

**Affiliations:** 1 Department of Nutrition, Faculty of Medical and Health Sciences, University of Auckland, 85 Park Rd, Grafton, Auckland, 1142 New Zealand; 2 Department of Speech Science, School of Psychology, University of Auckland, Auckland, New Zealand

**Keywords:** Puree, Dysphagia, Aged care, Hydrolysed meat, Enrichment, Texture-modified diets, Malnutrition

## Abstract

**Background:**

As a result of the high prevalence of dysphagia in aged care facilities, demand for pureed diets is increasing. One of the biggest challenges for pureed diets is the reduced nutritional density due to the cooking process, such as when blending or softening with liquid. This study aimed to investigate the impact of innovative energy and protein-enriched meat puree on the nutrition intake and nutritional status of aged care residents requiring pureed diets.

**Methods:**

This is a single-blinded randomised controlled trial conducted in two aged care facilities using a crossover design. Twenty-two residents aged 83.2 ± 7.3 years participated in a 12-week study. Participants were blocked randomised into two groups and received a 6-week of either control (unaltered freshly made pureed diets by facilities) or intervention diet, followed by a 2-week washout and then 6-week of alternative treatment. During the intervention, freshly made meat pureed portions were swapped to hydrolysed meat, which contained 144 -392 kcal and 5.6–6.8 g more energy and protein per 100 g. Nutrition intake was collected using a validated visual estimation method over 24 h on two non-consecutive days during the control and intervention phases. A two-tailed t-test was used to compare the significance.

**Results:**

The intervention diet significantly increased energy (147 ± 285 kcal, *p* = .02), protein (4 ± 7 g, *p* = .04), and fat (3 ± 8 g, *p* = .07) intake in comparison to the control diet. Nutritional status was improved by the end of the intervention as evidenced by a higher nutritional assessment score using Mini-Nutritional Assessment – Short Form (9.1 ± 1.8) and a weight gain of 1.3 ± 1.7 g, *p* = .04. No significant differences were found in body composition using bioelectrical impedance analysis, calf circumference and mid-upper arm circumference. Though handgrip strength did not differ at the end of control and intervention, significance was found between the changes in control and intervention period. Plasma branched-chain amino acid increased significantly with hydrolysed meat consumption.

**Conclusions:**

As a dietary enrichment, hydrolysed meat is a promising intervention for pureed diet consumers in aged care facilities, improving residents’ dietary intake and reducing malnutrition risk. Future larger multicentre studies with longer intervention periods are required to confirm the effectiveness and residents’ acceptance.

**Trial registration:**

Australian New Zealand Clinical Trials Registry (ACTRN12622000888763).

**Supplementary Information:**

The online version contains supplementary material available at 10.1186/s12877-022-03622-2.

## Background

Dysphagia is becoming more prevalent among older adults, and it will continue to be challenging as the demographic ages [1–3]. In comparison to hospitalised and community-dwelling older adults, the prevalence of dysphagia is higher in institutions, with 40–74% of aged care residents experiencing dysphagia [3–6]. Additionally, individuals with dysphagia are prone to weight loss and nutritional disorders as a result of chewing and swallowing disturbances [7]. Dysphagia is commonly seen in patients with neurological disorders, such as stroke, dementia and Parkinson’s disease [3]. Sarcopenia and malnutrition are also linked to dysphagia as a result of muscle weakness and loss of muscle mass. A high incidence of malnutrition is a major health concern in many long-term care institutions, and the common contributors recognised in research are poor oral intake, decreased appetite, chewing and swallowing problems and mealtime dependency [8]. The co-existence of dysphagia and malnutrition has been reported in both acute and institutional settings [6, 9]. Based on a previous systematic review, 3–28% of aged care residents experienced both dysphagia and malnutrition [5]. Dysphagia and malnutrition can both lead to other complications, increasing the likelihood of hospitalisations, cost of care, morbidity and mortality [10–12]. Therefore, nutritional management plays a crucial role in dysphagia care, particularly in older adults.

Texture-modified diets (TMDs) are the most common compensatory intervention for people with chewing or swallowing difficulties [13]. The texture and consistency of solids and liquids are modified to reduce swallowing effort and improve swallowing safety [14]. Over one-third of aged care residents consume TMDs, of which nearly half required pureed diets [8, 15–17]. Pureed diets require minimal chewing effort and are often prescribed to people with severe dysphagia because of the smooth texture and homogenous consistency. Our previous work identified the association between nutritional status and the level of TMDs in aged care residents [15]. Residents receiving pureed diets were more vulnerable to compromised nutritional status and often required oral nutritional supplements (ONS), as evidenced by low body mass index (BMI) and nutritional assessment scores [15, 16]. Several studies investigating the nutrition intake of aged care residents consuming pureed diets have shown inadequate energy and protein intake [18–20]. A recent review indicates that freshly made pureed diets have inconsistent nutrient levels and lower nutrient density than regular diets [7]. While the recently published International Dysphagia Diet Standardisation Initiative (IDDSI) provides a common definition and description of pureed diet, the nutrition perspective is still an established challenge faced by many healthcare providers [15, 17].

Recent research on pureed diets in aged care facilities has focused on using shaped or fortified meals [7]. Although several studies proved that shaped pureed diets could positively enhance dietary intake and body weight through improved appearance, shaping requires more preparation time and increased staffing [19, 21–23]. Alternatively, dietary intake can be optimised by offering denser nutrient content foods. In order to improve the nutrient density, pureed foods are often fortified with dairy products, protein powder, infant cereals and vitamin powder [22, 24–26]. Nutrition intake can be improved through fortification; however, some ingredients used in the process may cause a thickening effect or subtle change of taste [24, 25]. There is a growing body of literature investigating novel food processing technologies that can preserve better nutrients, aesthetics and flavour of pureed foods [27, 28]. Enzymatic treatment has been proposed as one of the conventional technologies that can soften traditional foods and has been applied to meat products [28]. Japanese researchers found that infusing hydrolytic enzymes can soften foods without alternating the appearances, and enzyme-infused pureed diets can significantly improve energy and protein intake [28–30]. Meat protein can be proteolyzed (a peptide bonds hydrolysis reaction) by plant proteases (enzymes) and lead to a higher protein solubility, dispersibility and emulsification [31]. Protein hydrolysis has been therefore used in special food formulations to meet special nutritional needs and improve palatability [32]. Despite the proven benefits of hydrolysed protein, the nutrition implication on pureed diet consumers has not been investigated. Based on our previous study, we found that main dishes made with hydrolysed meat contained significant higher protein content (17 g/100 g) compared to the same pureed dish prepared by foodservice (6 g/100 g) [33]. To tenderise the meat protein and create a smooth pureed texture, raw beef or chicken was hydrolysed by kiwifruit enzymes [34].

Considering older people have a higher protein need, in particular those with compromised nutrient metabolism caused by medical conditions, protein-dense purees would be beneficial to the aged care population [35]. In response to the nutritional challenges among long-term pureed diet consumers, the purpose of this study was to examine the impact of hydrolysed meat on the dietary intake and nutritional status of aged care residents. We hypothesised that the 6-week intervention diet consisting of hydrolysed meat may result in greater dietary intake and nutritional status improvement compared to the 6-week control diet consisting of ‘usual care’ cook-fresh pureed meat.

## Methods

This block randomised crossover trial was conducted at two aged care facilities in Auckland, New Zealand. The crossover design was chosen to minimise the risk of confounders in this vulnerable group who were likely to have multiple chronic conditions [36]. The participants served as their own controls, which allows us to compare the outcomes over time for the same participants. Ethics approval for this study was received from the Northern A Health and Disability Ethics Committees on May 11, 2021 (21/NTA/33). The study was designed and reported following the Consolidated Standards of Reporting Trials (CONSORT) checklist and was registered at the Australian New Zealand Clinical Trials Registry on 22/06/2022 (ACTRN12622000888763).

### Participants and recruitment

Email invitations were sent to eligible aged care facilities located in Auckland. Facilities were considered eligible if they offered both hospital and rest-home levels of care and provided freshly made pureed diets. Two medium aged care facilities (occupancy = 52 and 60) belonging to the same organisation voluntarily participated in the study. Both facilities were in the urban area, and all meals were freshly cooked in their kitchen. Facility managers provided written consent.

After gaining the permission of facility approval, consenting managers were asked to provide a list of eligible residents who are consuming pureed diets. Residents were eligible for inclusion if they were aged over 65 years old and received pureed diets on a daily basis. Exclusion criteria were respite or palliative care, receiving external fluids (enteral or parenteral feeds), a partially pureed diet, or being unable to consume chicken and beef. The participant information sheet was sent to all eligible participants and their next of kin. Written informed consent from each participant and their supportive family members was obtained.

### Sample size

Compared to a parallel randomised controlled trial, a crossover design requires less study participants [37]. The sample size was calculated based on detecting the weight change after six weeks of intervention from a previous study where pooled published data revealed a 0.7 kg body weight increase after six weeks of protein-enriched and shaped TMDs in 16 aged care residents [22]. In order to achieve the power of 0.8 for two-tailed t-tests, a sample size of 21 was required to enable the detection of 0.5 kg body weight change for a medium effect size α = 0.05. Our study aimed to recruit 26 participants to allow 20% drop-out.

### Randomisation and blinding

**Fig. 1:**
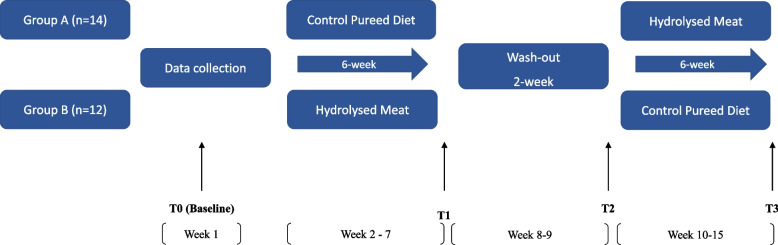
Study design. Only the protein component differed between control and intervention. Pureed hydrolysed beef/chicken was offered at lunch and dinner instead of the usual pureed protein in control diet. Other components in pureed meals were the freshly made carbohydrate and vegetable portions based on daily menus. T0 = week 1, T1 = week 7, T2 = week 9, T3 = week 15

**Fig. 2:**
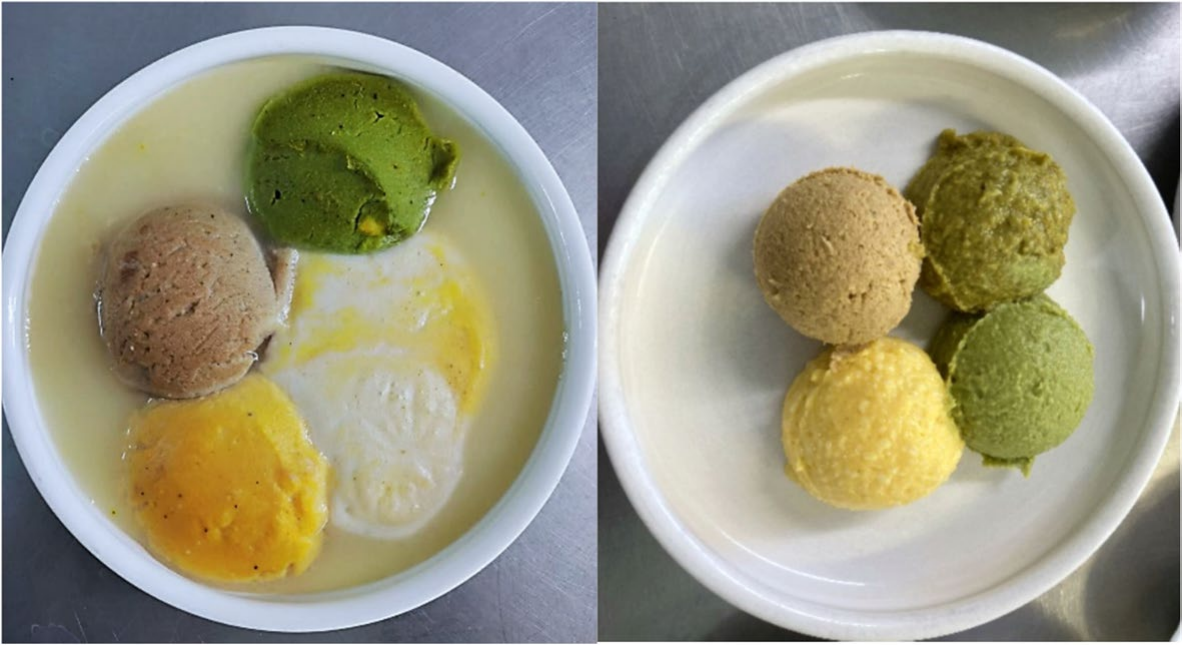
Comparison of the apperances of intervention diet and control diet. Intervention diet (left) with hydrolysed meat (brown portion). The control diet (right) with freshly made pureed meat (brown portion)

This study used a single-blinded block randomised crossover design with a 6-week intervention (hydrolysed meat)/control (unaltered freshly made pureed meat), a 2-week washout and a 6-week control/intervention (Fig. 1). Each facility was treated as a block and received either the intervention or the control diet in a random order. During the 2 weeks of washout period, participants did not receive any intervention and continued their usual pureed diet provided by their facility. After the washout period, they received the opposed treatment for another 6 weeks. Participants were allocated to one group (A or B) based on the facility, with all participants recruited from one aged care facility randomly allocated in group A, and all participants in the other facility randomly allocated in group B. Computer randomisation software was used to complete the block randomisation. There was knowledge of these allocations among researchers and foodservice staff who were responsible for meal preparations. The presentation of hydrolysed meat and freshly made pureed meat have no significant differences (Fig. 2). Participants and other staff were blinded to the intervention. The student dietitians collecting dietary intake data were also blinded to the study design. The research team including the registered dietitian responsible for assessments were unblinded.

### Interventions

Both facilities served three main meals and three mid-meals with unaltered freshly made foods listed on a 4-week cycled menu. Meat was offered at lunch and dinner, and therefore, the intervention diet substituted the traditional pureed meat with the same quantity of hydrolysed meat (beef or chicken) twice a day. All pureed meals were plated in the kitchen by chefs using standardised ice cream scoops and then delivered to residents by healthcare assistants. One scoop of puree was considered a standard serving size. Diet typically included one portion of pureed meat, two portions of pureed vegetables, one portion of pureed carbohydrate, and gravy.

Portion sizes were individualised based on resident preferences and all participants were offered at least one scoop of meat portion. For participants requiring larger portion sizes, two scoops of meat and/or vegetables were served. Hydrolysed meat was pre-packed into 1 kg per pack and delivered to the facilities. It was processed with 80% lean beef or whole blended chicken, salt, pepper, buffed vinegar, citrus fibre, water and kiwifruit enzyme. Flavours, such as spice, herbs and sauce, can be added to hydrolysed meat based on foodservice daily recipes. Previous consumer testing by dietitians and speech-language therapists demonstrated that hydrolysed meat was suitable for pureed diet consumers and compliant with IDDSI standards [33]. The hydrolysed meat was ready-to-eat and can be kept in the fridge for up to 90 days. Table 1 compares the nutrition content between hydrolysed meat and usual freshly made purees by the foodservice. Energy and protein were higher in hydrolysed meat purees due to the higher proportion of meat and less water content. Foodservice staff was trained by the research dietitian to prepare, heat up and store the hydrolysed meat. During the intervention period, chefs continued to prepare pureed diets using their regular recipes but replaced the usual raw meat with hydrolysed meat. Dietary changes were made only to the meat component between the control and intervention periods. The group receiving the control diet continued with their usual freshly made pureed diet according to the weekly menu. Participants maintained their usual dietary habits throughout the study, including the usual meal routine and ONS administration.

**Table 1:** Nutrition content of hydrolysed meat and usual freshly made meat purees (per 100 g) used in the study

	Hydrolysed beef (/100 g)	Freshly made beef puree (/100 g)	Hydrolysed chicken (/100 g)	Freshly made chicken puree (/100 g)
Energy (kJ)	1030	638	641	497
Protein (g)	20.8	14	15.4	9.8
Total fat (g)	17.8	10.0	9.5	8.2
Saturated fat (g)	7.4	3.3	2.7	2.1
Carbohydrate (g)	1.8	1.5	1.8	1.6
Sodium (mg)	421	368	440	360

Hydrolysed beef/chicken was made of 80% lean beef or whole blended chicken, salt, pepper, buffed vinegar, citrus fibre, water and kiwifruit enzyme. Freshly made beef/chicken purees were made of boiled beef fillet/whole chicken without skin, potato, canola oil, stock and salt

### Outcome measurements

Nutrition intake data were collected throughout the study by a group of master’s student dietitians, while other measurements were completed by a registered dietitian researcher at four-time points: baseline (week 1- T0), the end of the first phase of treatment (week 7 -T1), the end of washout (week 9—T2) and the end of the second phase of treatment (week 15, T3). Data collection for this study was carried out from May 17th to August 29th, 2021.

### Participant characteristics

Demographic information was collected from their medical records at week 1, including gender, age, ethnicity, admission date, level of care, medical conditions, mobility level, pressure sores, bowel movement frequency, reasons for pureed diet prescription, thickened fluids (TFs) requirement and ONS requirement. Clinical managers verified the collected information to ensure that it was accurate. Barthel Index (BI) questionnaire was used to assess participant functional capacity levels for basic activities of daily living, including personal care and mobility [38]. Dependency severity was measured via an ordinal scale of 0 to 100 points, where the lower scores represent higher dependency. Gastrointestinal symptoms and adverse events were monitored by the nursing staff as part of their clinical routines. Staff were asked to notify the research team of any remarkable clinical changes.

### Dietary assessment

To increase the reliability of habitual dietary intake, nutrition intake was assessed on two non-consecutive days each week throughout the study except during the washout period (week 8–9). Mean intake for each period was calculated over two days in baseline, twelve days in the first and twelve days in the second phase. All foods and drinks served to participants over 24 h were measured, including main meals, mid-meal snacks, ONS and beverages. Recipes and meal components were provided by chefs. The dietary intake was not collected if participants dined outside of the facility. Academic dietetic supervisors trained twenty final-year master’s student dietitians to use the visual estimation method to conduct observations of dietary intake. Each student conducted 2–3 days of mealtime observation in one facility and recorded the recipes, standard serving size measurements and plate wastage estimation on a password-protected shared drive for further analysis. If participants consumed any foods or drinks after supper and before breakfast, healthcare assistants would record the details and inform the student the following day. Photos of foods were taken before and after consumption to minimise bias in food waste estimation and allow researchers to validate the accuracy.

The visual estimation method has been used in several studies and is a validated tool for measuring plate wastage of pureed diets [15, 39–41]. All foods and drinks were weighed twice to the nearest 1 g using standard electronic kitchen scales (Salter High-Capacity Scales, Model 1160 BKDR) the first time served at each home and recorded as a standard serving size. Once participants finished eating, students used a 7-point scale to visually estimate the amount of each food item has been left on the plates (‘all left (100%)’, ‘mouthful eaten (90%)’, ‘3/4 left (75%)’, ‘1/2 left (50%)’, ‘1/4 left (25%)’, ‘mouthful left (10%)’ and ‘none left (0%)’) [39]. The amount of food eaten was then calculated by multiplying the percentage of consumption and the standard serving size. Plate wastage surveys (Appendix 1) were completed each data collection day. To determine the nutrient levels, recipes and dietary intake were then entered in FoodWorks 10 (Xyris Software; Brisbane, Australia).

### Nutritional assessment

The Global Leadership Initiative on Malnutrition (GLIM) is recommended for assessing nutritional status in patients with dysphagia, which includes 1) a nutritional screening tool, 2) phenotypic characteristics (weight/BMI/muscle mass change), and 3) etiologic changes (dietary intake/blood biomarkers) [42]. Based on previous literature, Mini Nutritional Assessment Short Form (MNA-SF) is a validated nutrition screening and assessment tool and has been widely used in long-term care [43–45]. Therefore, the MNA-SF was used for the nutritional assessment in this study. The dietitian researcher collected information from clinical records, and clinical staff were asked for any missing information. Scores under 8 are considered malnourished; 8 to 11 indicates at risk of malnutrition, and 12 to 14 indicates normal nutritional status [46].

### Body weight, calf circumferences, mid-upper arm circumferences

Body weight was taken by the facility nurses using the chair scale after the morning shower on the last day of each period. To ensure weights were collected accurately, using the same chair scale with minimal clothing was the most plausible method. We were unable to assess the standing heights in immobile participants, therefore, ulna length was used in this study as a recommended alternative measurement to estimate the height of older adults [47]. BMI was then determined using weight (kg) divided by height (m^2^). Examinations of the non-dominant calf and mid-upper arm circumferences are indicators of muscle mass and malnutrition [48, 49]. Measurements were taken following the anthropometric guidelines using a dedicated tape; less than 31 cm calf circumferences and 23.5 cm MUAC were recognized as lower muscle size and likely to be underweight [50, 51].

### Handgrip strength (HGS)

An assessment of HGS is a practical indicator of overall muscle strength and function, and is associated with nutrition intake [52, 53]. HGS of the dominant limb was measured using a JamarTM Hand Dynamometer. Participants were in a seated position and asked to rest their forearms on the arm of the chair, with shoulders abducted in neutral rotation and forearms flexed at a 90-degree angle. They were asked to position their thumb around one side and their fingers around the other side of the handle. For those who were unable to sit up straight, the measurement was taken in a supine position. The researcher encouraged the participant to squeeze as hard as possible and took three consecutive measurements with a 1-min rest interval. The hand dynamometer handle length was optimised for the participant. The maximum value was recorded to the nearest 1 kg.

### Body composition

Bioelectrical Impedance Analysis (BIA) is a non-invasive and convenient method to estimate body composition via measuring the differences in electrical resistance and reactance of body tissues [54]. The trained researcher performed the measurements using a single-frequency instrument (Bodystat 1500 MDD). To ensure the hydration status is standardised and results were reproducible, BIA was conducted in the morning before breakfast and after emptying the bladder. We followed the Standard Operating Procedures for using the Bodystat 1500 v4 published by National Institute for Health Research Southampton Biomedical Research Centre. Participants were asked to lie in the supine position for 5 min before taking the measurements. Two pairs of electrodes will be placed, one pair on the dorsum of the hand and another pair on the dorsum of the foot, with each electrode at a 5 cm distance. Following the measurements, the data was imported to Body Manager Pro (Bodystat Ltd; Douglas, Isle of Man) for body composition analysis, including phase angle, lean mass, fat mass, total body water, and intra- and extra-cellular water. Phase angle was the outcome extracted from the BIA measurement, which is considered a useful prognostic indicator of health and nutritional status. Phase angle is positively associated with cell capacitance (membranes) and negatively associated with resistance (fluid), and thus higher phase angle reflects healthier cellular integrity and thriving cellular health [55].

### Biochemistry

After discussing with clinical managers, participants and their families, we recognised the challenges of blood specimen collection in this vulnerable group, such as physical impairment (skin changes, fragile blood vessels, tremors, rigidity), psychological stress and higher risk of bruising [56]. Therefore, fasting blood specimen collections were only taken at the end of two phases (T1 and T3) by experienced geriatric phlebotomists. Blood samples were centrifuged at 2000 rpm for 15 min at 4 °C and then frozen at -80 °C until analysis. Serum c-reactive protein (CRP), serum albumin and plasma insulin-growth factor -1 (IGF-1) were obtained as indicators of inflammation and have been used to provide an overall picture of nutritional status [30, 48]. To explore if the higher protein content in hydrolysed meat would lead to greater plasma amino acid availability, plasma amino acid analysis was performed using ultra-performance liquid chromatography in accordance with previously published methods by trained laboratory technicians [57]. Specifically, levels of the total amino acid, branched-chain amino acid (isoleucine, leucine, valine), essential amino acid (histidine, isoleucine, leucine, lysine, methionine, phenylalanine, threonine, tryptophan, and valine) and collagen peptide-derived amino acid (glycine, hydroxyproline, proline) were compared between control and intervention diet.

### Statistical methods

Data management and analysis were performed using GraphPad Prism v9.2 (GraphPad Software, Inc., San Diego, CA, USA) for statistical analysis. We conducted Shapiro–Wilk tests to determine the normality of all variables. Normally distributed continuous variables are presented as mean with standard deviation (SD), and median with interquartile range [IQR, 25-75th percentile] was used for non-parametric variables. Categorical variables are expressed as the count and percentage of the total (%). Two-tailed paired t-tests were conducted to compare the outcome changes between study phases. Data were analysed as intention-to-treat (all participants with completed baseline assessments were included), and missing data were handled with multiple imputations [58]. A *p*-value of less than 0.05 was considered statistically significant.

## Results

### Participant characteristics

Twenty-seven aged care residents requiring pureed diets were assessed for eligibility and five were excluded: palliative care (*n* = 1), not complied to a full pureed diet (*n* = 1) and declined to participate (*n* = 3). A total of 22 participants aged 83.2 ± 7.3 years were randomised and included in the analysis, with 11 in group A and 11 in group B. Two participants were lost to follow-up due to death and one left the facility. Participant characteristics and Barthel Index scores are summarised in Table 2. More than half of the participants required routine ONS and 45% required TFs. None of the participants had pressure sores. Dementia was the most common diagnosis (*n* = 15, 68%), followed by hypertension (*n* = 12, 55%), stroke (*n* = 7, 32%), depression/anxiety (*n* = 6, 27%), diabetes (*n* = 5, 23%), osteoarthritis (*n* = 5, 23%) and chronic obstructive pulmonary disease (*n* = 4, 18%), Two participants with a diagnosis of Parkinson’s disease, and one had Huntington’s disease. No adverse events such as gastrointestinal symptoms, allergy reaction, aspiration, or choking occurred while the study was conducted. Similarly, no significant changes in bowel movement were noted during the study.

**Table 2:** Participant characteristics at baseline (*n* = 22)

Variables	n (%) / Mean (SD)	**Median [IQR]**
Age (years)	83.2 (7.3)	85 [81, 87]
Gender	Female – 12 (55%) Male – 10 (45%)	
Ethnicity	Chinese – 10 (45%) NZ European – 8 (36%) Other European – 2 (9%) Maori – 2 (9%)	
Level of care	Hospital – 18 (82%) Rest home – 4 (18%)	
Average length of stay (months)	32.4 (28.7)	24.1 [6.7–58.1]
Mobility level	Independent – 5 (23%) Semi-independent – 6 (27%) Wheelchair – 3 (14%) Bedridden – 8 (36%)	
Feeding requirement	Full assistance – 13 (59%) Independent – 9 (41%)	
No. of medical conditions	5.3 (2.5)	5.0 [3.5–7.5]
Bowel movement frequency per week	< 3 – 3 (14%) 3–7 – 14 (64%) 8–14 – 5 (23%)	
Use of laxative	17 (77%)	
Reasons requiring pureed diet	Dysphagia diagnosed by a speech-language therapist – 12 (55%) Chewing difficulty noted by a nurse– 4 (18%) Eating & drinking difficulties due to dementia noted by a nurse – 6 (27%)	
Oral nutritional supplement	15 (68%)	
Thickened fluid requirement	10 (45%)	
Barthel Index score (out of 100)	22.9 (29.9)	5.0 [0.0–45.0]

Continuous variables are presented as mean

Categorical variables are presented as count (percentage of total participants in the study)

*SD* Standard deviation and median, *IQR* 25-75th interquartile range

### Nutrition intake

Hydrolysed meat consumption resulted in significantly higher daily energy and protein intakes (Table 3). Intake increased across all macronutrients and sodium during the intervention period. There were no significant differences in nutrition intake between baseline and control diet. One participant went back home during the wash-out period and therefore the food intake was not accounted for in those two days.

**Table 3:** Comparison of mean (SD) daily nutrition intake between freshly made meat purees and hydrolysed meat (*n* = 22)

Nutrient	Baseline	Control	Intervention	Differences^a^	*p*-value
Energy (kcal)	1556 (303)	1516 (324)	1663 (299)	+ 147 (285)	**.02**
Protein (g)	66 (15)	68 (14)	72 (14)	+ 4 (7)	**.04**
Total fat (g)	57 (15)	53 (12)	56 (12)	+ 3 (8)	**.07**
Saturated fat (g)	24 (5)	24 (5)	25 (6)	+ 1 (4)	.14
Carbohydrate (g)	181 (36)	182 (51)	167 (30)	-15 (47)	.15
Sodium (mg)	1901 (476)	1757 (414)	1815 (429)	+ 58 (561)	.63

Baseline data were collected throughout two non-consecutive days in week 1. Control and intervention data were both collected through two non-consecutive days over six-week period. ^a^Differences were calculated by subtracting control value from intervention value. Paired t-tests were used to test the significance between control and intervention

*P* < .05 is considered significant

### Nutritional status, muscle functionality and body composition

At baseline, 36% (*n* = 8) of the participants were malnourished and 55% (*n* = 12) were at risk of malnutrition. An improvement in nutritional status was found in 45% (*n* = 10) of the participants post-intervention. Table 4 summarises the changes in nutritional status, body weight, body composition and muscle strength. Weight gain (0.9 ± 1.4 kg) and HGS improvement (0.5 ± 1.6 kg) were observed during the intervention period, while weight loss (-0.3 ± 1.5 kg) and HGS reduction (-1.9 ± 1.6 kg) occurred during the control period. MNA-SF scores and BMI were significantly higher with the intervention diet as a result of weight improvement. There were no evident changes in muscle mass or body composition over the course of the study. Due to muscle rigidity, advanced cognitive and communication impairment, many participants were unable to complete the HGS tests. Only eight participants were able to complete the HGS tests following the correct instruction. Similarly, four participants were unable to adjust their bodies or limbs to the supine position required for BIA. To ensure data consistency, we adapted them to the closest possible standard position and conducted the four time-point measurements using the same position.

**Table 4:** Changes of nutritional status, muscle functionality and body composition in mean (SD) (*n* = 22)

Outcomes	Baseline	Control	Intervention	*p*^a^-value	Changes at control	Changes at intervention	*p*^b^-value
MNA-SF	8.3 (2.4)	8.3 (2.2)	9.1 (1.8)	**.04**	-0.7 (2.7)	+ 0.9 (1.4)	**.03**
Weight (kg)	52.3 (13.1)	52.5 (13.9)	53.6 (13.5)	**.004**	-0.3 (1.5)	+ 1.3 (1.7)	**.004**
BMI (kg/m^2^)	19.9 (3.7)	19.9 (3.6)	20.4 (3.5)	**.008**	-0.1 (0.6)	+ 0.5 (0.7)	**.005**
CC (cm)	28.9 (4.6)	28.3 (4.7)	28.6 (4.4)	.41	-0.04 (1.8)	-0.3 (1.4)	.69
MUAC (cm)	25.1 (4.1)	24.6 (3.4)	25.0 (3.3)	.29	-0.3 (2.1)	-0.04 (2.1)	.60
^a^HSG (kg)	18.0 (5.7)	18.3 (9.3)	18.5 (4.7)	.90	-1.9 (1.6)	+ 0.5 (1.6)	**.03**
Phase angle ^o^	3.9 (0.8)	4.6 (1.5)	3.9 (1.0)	.12	+ 0.7 (1.8)	+ 0.04 (0.9)	.12
% Fat	33.1 (10.3)	35.8 (11.0)	33.3 (11.4)	.37	+ 1.8 (12.0)	+ 0.2 (10.5)	.66
% Lean mass	66.9 (10.3)	66.0 (12.1)	66.7 (11.4)	.37	-1.8 (12.0)	-0.2 (10.5)	.66
% Water	64.5 (8.5)	64.2 (9.7)	64.0 (8.4)	.65	-0.9 (7.1)	-0.5 (6.4)	.85

Both control and intervention period were 6 weeks. Changes at control/intervention = the end of control/intervention T1/T3—baseline T0/the end of washout T2 depending on the group allocation.

*MNA-SF*  Mini Nutritional Assessment Short-form, *BMI* Body Mass Index, *CC*  Calf Circumferences, *MUAC*  Mid-Upper Arm Circumferences, *HSG* Handgrip strength

*p*^a^ = differences between control and intervention values using parried t-test;

*p*^b^ = differences between changes during control period and changes during intervention period using paired t-tests. *P* < .05 is considered significant

^a^Only eight participants were able to follow the instructions and complete handgrip strength test due to muscle rigidity, cognitive and communication impairment

### Biochemistry

Biochemistry results were analysed from 13 blood samples (Table 5). Blood specimen collections were unavailable from nine participants due to refusal and emotional or physical discomfort. There were no significant differences were observed in CRP, Albumin and IGF-1. The results were normally distributed except for CRP, which had two abnormal results due to acute conditions. Total amino acid, essential amino acid and collagen peptide-derived did not differ by treatment. However, the branched-chain amino acid was 16% greater with the 6-week consumption of hydrolysed meat (*p* = 0.007).

**Table 5:** Biochemistry results measured at the end of control and intervention diet in mean (SD) or median [IQR] (*n* = 13)

Outcomes	Control	Intervention	*p*-value
CRP (mg/L)	5.0 [3.0—10.0]	4.0 [3.0 – 13.0]	.63
Serum Alb (g/L)	33.3 (1.9)	32.5 (2.6)	.18
Plasma IGF-1 (μg/L)	97.8 (20.4)	97.2 (17.2)	.88
TAA (μmol/L)	2862.0 (326.2)	2893.1 (301.9)	.80
EAA (μmol/L)	844.2 (99.0)	889.6 (151.5)	.38
BCAA (μmol/L)	329.8 (37.4)	383.0 (62.1)	**.007**
Collagen-peptide derived AA (μmol/L)	489.9 (105.5)	485.5 (82.4)	.84

*CRP* C-Reactive Protein (reference value 0–5 mg/L), *Alb* Albumin (reference value < 35 g/L);), *IGF-1* Insulin Growth Factor-1 (reference value 21–206 μg/L for females ≥ 75 yr; 22–204 μg/L for males ≥ 75 yr), *TAA* Total Amino Acid (*n* = 23); *EAA* Essential Amino Acid (histidine, isoleucine, leucine, lysine, methionine, phenylalanine, threonine, tryptophan, and valine), *BCAA* Branched-Chain Amino Acid (isoleucine, leucine, valine),* Collagen-peptide derived AA* Collagen-peptide derived Amino Acid (glycine, hydroxyproline, proline). Parametric variables are presented as mean (*SD* standard deviation) and tested with paired t-test. Non-parametric variables are presented as median [*IQR* 25-75th interquartile range] and tested with Wilcoxon matched-pairs signed rank test.

*P* < .05 is considered significant

## Discussion

The present crossover randomised controlled trial explores, for the first time, the impact of hydrolysed meat with high energy and protein composition among aged care residents requiring pureed diets. The 6-week implementation of hydrolysed meat successfully improved residents’ nutrition intake, nutritional status and plasma amino acid concentrations. Participant demographics were similar to those reported by previous TMDs intervention studies conducted in aged care facilities [15, 19, 22, 23, 26, 59, 60]. Based on a cross-sectional study conducted in Chinese aged care facilities, the average dependency level of aged care residents with dysphagia was significantly lower than those without dysphagia, with an average of 24.6 ± 30.8 out of 100 [61]. Our results showed a similar average dependency score using Barthel Index (22.9 ± 29.9) but an extremely low median score (5.0), indicating the severe dependency in activities of daily living of these pureed diet consumers. Additionally, we confirmed that dementia was the most common diagnosis, corroborating published data that a large proportion of residents requiring pureed diets are cognitively impaired and in need of feeding assistance [15, 18, 19, 22, 59, 61]. Residents with severe dementia are more likely to experience deteriorating dental health and lower BMI [62]. Energy-enriched meals have a greater impact on lower BMI residents, as they consume significantly more energy and protein [63].

### Nutrition intake

The nutrition intake at baseline and during the control period is comparable with previous studies assessing the intake of residents consuming traditional freshly made pureed diets (energy: 908–1662 kcal/day and protein: 42-68 g/day) [20, 26, 60, 64–67].

Our study demonstrated a significant 10% increase in energy intake and a 6% increase in protein intake with our intervention of hydrolysed meat. Previous work shows similar findings, with Jones et al. reporting a significant 12% increase in energy intake (225 kcal/day) with energy-enriched pureed meals in healthy adults without affecting the palatability, satiety and appetite [68]. The positive increase in energy and protein intake in our study is in agreement with findings obtained in studies that incorporated fortification and ONS for pureed diets [22, 26, 65]. Previous studies found that 59% to 74% of aged care residents could consume a full pureed meal [17, 66]. Older adults tend to have a small appetite, particularly those with low physical activity levels. By providing meal portions tailored to residents’ usual dietary habits, we were able to determine whether hydrolysed meat was beneficial to nutrition intake without increasing the quantity served. The positive results in dietary intake demonstrate that successful nutrition intervention could be achieved by substituting one component with a nutrient-dense alternative without increasing the volume of food.

### Nutritional status

Vucea et al. investigated the characteristics of pureed diet consumers in 32 aged care facilities and reported that residents prescribed with unmodified pureed diets had a 0.9 ± 4.1 kg weight loss over three months and an average BMI of 19.1 ± 13 kg/m2 [16]. Consistent with their study, our participants had 0.3 ± 1.5 kg weight loss over one and a half months when consuming the unmodified freshly made puree diets. Meanwhile, a significant weight gain over the 6-week intervention was observed. The amount of energy and weight increase are consistent with previous research involving malnourished residents, which reported a 133 kcal/day higher energy intake and a 1.3 kg weight gain with 12 weeks of energy-enriched diet [69]. Similarly, a study using fortified purees and ONS in aged care demonstrated a successful effect on energy intake (48% increase) and weight gain after six months (4.6 ± 2.0 kg) [26]. Ott and colleagues have been unable to demonstrate the weight differences between 6-week traditional TMDs and 6-week enriched and shaped TMDs, though weight changes between the two treatment periods were significant (0.5 kg weight loss over control and 1.1 kg weight gain over intervention) [22]. These findings provide support for the weight gain seen with our intervention.

The MNA-SF scores, calf circumferences and MUAC are in line with those reported in previous studies [15, 44, 70]. MNA-SF scoring is challenging in aged care, where residents often score low due to reduced mobility, psychological stress and dementia [15, 71]. There were no changes in mobility and neuropsychological problems during our study, and therefore, changes in the MNA-SF score were mainly due to the variations in weight and food intake. The decreased calf circumference confirmed the high risk of malnutrition. While calf circumference and MUAC are recommended measurements in older adults as indicators of nutritional status and associated with mortality, significant changes may occur over a more extended period in a very sedentary and bedridden population [49, 72]. There was no convincing evidence indicating muscle changes in our study.

Similarly, Leslie et al. did not find changes in MUAC with an enriched diet despite the weight gain [69]. We are the first study to include body composition analysis using BIA in aged care residents with dysphagia. The BIA results seem consistent with other research that has measured the body composition in older hospital patients with dysphagia [43, 73]. Although phase angle is a good indicator of muscle mass and has been used to predict mortality and sarcopenia, the cut-off value has not been determined [74]. Reyes-Torres et al. reported an increase in phase angle and HGS with 12 weeks of systematically modified TMDs in institute patients [73]. Participants in Reyes-Torres’ study were younger (75.5 years) and received swallowing rehabilitation, while our study participants were older, and the intervention time was half of their duration [73]. The baseline HGS results found in our study are similar to the findings by Reyes-Torres et al. and were both below the European Group for the Study of Sarcopenia in Older Adults reference point (27 kg for men, 16 kg for women), indicating low muscle strength [73, 75]. Sarcopenia is categorised as a geriatric syndrome, which can be caused by a complex mechanism and results in numerous adverse outcomes [75]. Reduced muscle mass, muscle strength and physical performance have been reported in older patients with dysphagia, which leads to a high prevalence of sarcopenia (20%) [43, 76]. Inadequate nutrition is one of the multiple contributing factors to sarcopenia. Our study did not assess the physical performance due to the low mobility level, however, the reduction in muscle mass and strength was in agreement with Carrión’s study [43]. Future studies should consider including sarcopenia assessment when assessing people with dysphagia.

### Biochemistry measurements

There was no significant difference in biological indicators of nutritional status at the end of control and intervention, suggesting no evidence of acute inflammation [43]. CRP, albumin and IGF-1 were used to test visceral protein concentrations to identify whether the protein was deficient, and the average results were all within the normal ranges [77]. Studies examining biomarkers in adult patients with dysphagia have primarily been conducted in acute settings, and albumin has been the most frequent biomarker used in nutritional assessment [42]. In an experimental study using high-quality personalised TMDs in aged care residents, researchers found a progressive improvement of serum albumin over the 6-month intervention, yet, significance was observed after four months [78]. Similarly, Welch et al. observed a 10 g/L increase in serum albumin after three months of ONS and results were maintained at six months. Accordingly, it is possible that a longer intervention or a greater number of participants would be necessary to observe the significance of aged care residents consuming pureed diets.

Levels of amino acids have been studied by Leibovitz et al. in residents with dysphagia and reported all within the normal range with an intake of 1560 ± 350 kcal energy and 57 ± 18 g protein, and 33 ± 4 g/L serum albumin [79]. Branched-chain amino acid obtained with the control diet is consistent with Leibovitz et al.’s findings which showed 329 ± 75 μmol/L branched-chain amino acid [79]. The significant increase in the branched-chain amino acid is possibly a response to the higher protein intake through hydrolysed meat [80]. Hydrolysed beef and chicken both provide high-quality animal protein with a particularly notable amount of leucine [81, 82]. Leucine is one of the three amino acids in the branched-chain amino acid, which can stimulate protein synthesis in skeletal muscle and build lean body mass [80]. A higher branched-chain amino acid may positively influence muscle strength, which was also found in our study, as evidenced by the significant changes in HGS between control and intervention diets [83].

In addition, plasma amino acids are influenced not only by protein consumption but also influenced by tissue synthesis [84]. People consuming different diet groups were more likely to show differences in plasma amino acid concentrations, such as different types of meat and vegan protein [85]. An extended period of intervention may be required to detect the significant changes in total amino acid and essential amino acid.

## Limitations

A strength of this study is that the crossover design offers equal treatment opportunities to all participants and minimises the between-subject variability, thereby increasing the precision of estimation. It is, however, not possible to generalise the results of this study to all aged care residents who require pureed diets due to the small sample size and participant characteristic variations. For example, the majority of the participants in our study had cognitive impairment and limited physical functionality. This in turn also led to missing data where residents were unable to complete all the assessment battery. Furthermore, our study did not conduct swallowing assessments to confirm participants’ swallowing ability and some of the participants were prescribed pureed diets by doctors rather than speech-language therapists. The number of residents with dysphagia may be underestimated.

The study was conducted in a cook-fresh foodservice operation, so findings may differ in other foodservice types. Due to the small number of residents requiring pureed diets in each facility, randomisation within the facilities was unable to achieve in this study. Despite both facilities being run by the same organisation, facility-level foodservice factors could have confounded the findings.

Participant acceptance and satisfaction were not assessed. Residents with dysphagia may have a decreased perception of texture, taste and smell and different acceptance of purees compared to healthy adults due to impaired cognition and age-related sensory degeneration [86]. Some of the clinical outcomes were insignificant in this study, thus, a multicentre randomised controlled trial with larger sample size and more extended experimental period is required to confirm the findings and reduce participant characteristic biases. *P* < 0.05 was chosen as a measure of statistical significance based on the exploratory nature of this study given the small sample size. However, multiple comparisons may arise as the results of involving multiple simultaneous statistical tests and thus, create difficulty in interpreting results. Our study did not discuss the financial costs and feedback from foodservice. Future studies should consider gathering insights of the implementation generalisability and feedback from participants and facility management.

## Conclusions

The results of this crossover randomised controlled trial concluded that hydrolysed meat could be successfully used as a high-energy and protein substitute for traditional freshly made meat purees. Nutrition intake and nutritional status can be improved via hydrolysed meat and other forms of nutrient-enriched pureed foods. Aged care residents requiring pureed diets often have dysphagia or dementia and are at high risk of malnutrition. Consequently, nutrition management should be prioritised to prevent malnutrition and other complications. Further larger-scale research is required to confirm the findings of our study and explore sustainability in long-term implications.

## Supplementary Information

**Additional file 1.**

## Data Availability

All data generated or analysed during the current study are included in this published article.

## Abbreviations

BIA: Bioelectrical Impedance Analysis
BMI: Body Mass Index
CRP: C-Reactive Protein
HGS: Handgrip strength
IDDSI: International Dysphagia Diet Standardisation Initiative
IGF-1: Insulin-Growth Factor -1
IQR: Interquartile Range
MNA-SF: Mini Nutritional Assessment Short Form
ONS: Oral nutritional supplements
SD: Standard Deviation
TMDs: Texture-modified diets
TF: Thickened fluids
