# Supplementary material for: Effects of hydrolysed meat on dietary intake and nutritional status in aged care residents requiring pureed diets: a crossover randomised controlled trial
Source: BMC Geriatr. 2022 Nov 25;22:905. doi: 10.1186/s12877-022-03622-2 (PMC9700874; doi:10.1186/s12877-022-03622-2)
Supplement: Supplementary file 1 — Additional file 1. [file 12877_2022_3622_MOESM1_ESM.docx]

**Plate wastage collection**

| **Participant ID:** | | **Date:** | | | **Week:** | | **Day: 1 2** | | |
| --- | --- | --- | --- | --- | --- | --- | --- | --- | --- |
|  | Quantity | All left 100% | Mouthful eaten 90% | 3/4 left  75% | 1/2 left  50% | 1/4 left  25% | Mouthful left 10% | None left 0% | Intake(g/ml) |
| **Breakfast items** |  |  |  |  |  |  |  |  |  |
|  |  |  |  |  |  |  |  |  |  |
|  |  |  |  |  |  |  |  |  |  |
|  |  |  |  |  |  |  |  |  |  |
|  |  |  |  |  |  |  |  |  |  |
| **Morning tea items** |  |  |  |  |  |  |  |  |  |
|  |  |  |  |  |  |  |  |  |  |
|  |  |  |  |  |  |  |  |  |  |
|  |  |  |  |  |  |  |  |  |  |
| **Lunch items** |  |  |  |  |  |  |  |  |  |
|  |  |  |  |  |  |  |  |  |  |
|  |  |  |  |  |  |  |  |  |  |
|  |  |  |  |  |  |  |  |  |  |
|  |  |  |  |  |  |  |  |  |  |
|  |  |  |  |  |  |  |  |  |  |
| **Afternoon tea items** |  |  |  |  |  |  |  |  |  |
|  |  |  |  |  |  |  |  |  |  |
|  |  |  |  |  |  |  |  |  |  |
|  |  |  |  |  |  |  |  |  |  |
| **Dinner items** |  |  |  |  |  |  |  |  |  |
|  |  |  |  |  |  |  |  |  |  |
|  |  |  |  |  |  |  |  |  |  |
|  |  |  |  |  |  |  |  |  |  |
|  |  |  |  |  |  |  |  |  |  |
|  |  |  |  |  |  |  |  |  |  |
| **Supper items** |  |  |  |  |  |  |  |  |  |
|  |  |  |  |  |  |  |  |  |  |
|  |  |  |  |  |  |  |  |  |  |
|  |  |  |  |  |  |  |  |  |  |
| **Other food/drinks** |  |  |  |  |  |  |  |  |  |
|  |  |  |  |  |  |  |  |  |  |
|  |  |  |  |  |  |  |  |  |  |
|  |  |  |  |  |  |  |  |  |  |

**Standard serving size measurement**

| **Sample meals** | **Date:** | | | **Week:** | | **Day: 1 2** |
| --- | --- | --- | --- | --- | --- | --- |
|  | Weight 1 (g/ml) | Weight 2 (g/ml) | Average weight (g/ml) | | Notes (e.g. quantity measured in cups/tbsp/tsps..) | |
| **Breakfast items** |  |  |  | |  | |
|  |  |  |  | |  | |
|  |  |  |  | |  | |
|  |  |  |  | |  | |
|  |  |  |  | |  | |
| **Morning tea items** |  |  |  | |  | |
|  |  |  |  | |  | |
|  |  |  |  | |  | |
|  |  |  |  | |  | |
| **Lunch items** |  |  |  | |  | |
|  |  |  |  | |  | |
|  |  |  |  | |  | |
|  |  |  |  | |  | |
|  |  |  |  | |  | |
|  |  |  |  | |  | |
| **Afternoon tea items** |  |  |  | |  | |
|  |  |  |  | |  | |
|  |  |  |  | |  | |
|  |  |  |  | |  | |
| **Dinner items** |  |  |  | |  | |
|  |  |  |  | |  | |
|  |  |  |  | |  | |
|  |  |  |  | |  | |
|  |  |  |  | |  | |
|  |  |  |  | |  | |
| **Supper items** |  |  |  | |  | |
|  |  |  |  | |  | |
|  |  |  |  | |  | |
|  |  |  |  | |  | |
| **Supplements** |  |  |  | |  | |
|  |  |  |  | |  | |
|  |  |  |  | |  | |
|  |  |  |  | |  | |
| **Other food/drinks** |  |  |  | |  | |
|  |  |  |  | |  | |
|  |  |  |  | |  | |

**Recipe**

| **Name of the food items** | **Ingredients** | **Weight of the ingredient (g)** |
| --- | --- | --- |
| Breakfast |  |  |
|  |  |  |
|  |  |  |
|  |  |  |
|  |  |  |
|  |  |  |
| Morning tea |  |  |
|  |  |  |
|  |  |  |
|  |  |  |
| Lunch |  |  |
|  |  |  |
|  |  |  |
|  |  |  |
|  |  |  |
|  |  |  |
| Afternoon tea |  |  |
|  |  |  |
|  |  |  |
|  |  |  |
| Dinner |  |  |
|  |  |  |
|  |  |  |
|  |  |  |
| Supper |  |  |
|  |  |  |
|  |  |  |
|  |  |  |
|  |  |  |
| Others |  |  |
|  |  |  |
|  |  |  |
